# Prevalence of Parasitic Infections with Zoonotic Potential in Tilapia: A Systematic Review and Meta-Analysis

**DOI:** 10.3390/ani12202800

**Published:** 2022-10-17

**Authors:** Víctor Johan Acosta-Pérez, Juan Carlos Ángeles-Hernández, Vicente Vega-Sánchez, Andrea Paloma Zepeda-Velázquez, Javier Añorve-Morga, Jesús Benjamín Ponce-Noguez, Nydia Edith Reyes-Rodríguez, Jorge Luis De-La-Rosa-Arana, José Gustavo Ramírez-Paredes, Fabián Ricardo Gómez-De-Anda

**Affiliations:** 1 Área Académica de Medicina Veterinaria y Zootecnia, Instituto de Ciencias Agropecuarias, Tulancingo de Bravo 43600, Mexico; 2 Área Académica de Química, Instituto de Ciencias Básica e Ingeniería, Kilometro 4.5 Carretera Pachuca—Tulancingo, Col. Carbonera Mineral de la Reforma, Hidalgo 42082, Mexico; 3 Medicina Veterinaria, Universidad de la Costa, Pinotepa Nacional, Oaxaca 71600, Mexico; 4 Microbiología en Salud Humana, Facultad de Estudios Superiores Cuautitlán, Universidad Nacional Autónoma de México, Cuahutitlan Izcalli, Estado de Mexico 54743, Mexico; 5 Ridgeway Biologicals Ltd., a Ceva Santé Animale Company, Units 1–3 Old Station Business Park, Compton RG20 6NE, UK

**Keywords:** aquaculture, fishing, host-parasite interactions, parasite load, tilapia, zoonotic parasites

## Abstract

**Simple Summary:**

The consumption of fish represents a healthy and affordable alternative for a large sector of the population; among the species with the highest consumption worldwide are tilapia. However, its consumption may be associated with health risks of a parasitic type. Through the analysis of the available scientific literature on parasitic prevalence in tilapia, it was observed that fish obtained from the wild and aquaculture represent a risk of parasitosis in human populations. Additionally, a high prevalence of cestodes and a greater diversity of trematodes were found. Finally, the presence of the genus *Gnathostoma* must stand out. This synthesis of information can be useful as a platform for the develCopment of intervention programs for parasite control, and it also suggests continuing with the study of zoonotic parasites related to the consumption of tilapia in geographical areas of high consumption.

**Abstract:**

Tilapia has a high socioeconomic value in many countries worldwide. However, it has been identified as a zoonotic parasite reservoir. A systematic literature search and meta-analysis were carried out in order to estimate the global prevalence of zoonotic parasites that affect tilapia. The search was performed by three field experts to avoid reviewer bias. Polled prevalence was estimated using a logistic-normal random-effect regression model in the R software. We dealt with the heterogeneity among studies through subgroup analysis, taking into account the continent, country, genus of the host, parasite taxonomic group, sample origin, and type of diagnostic test as moderator variables. Fifty-two eligible articles were identified covering five tilapia genera with a pooled prevalence of 0.14 (95% CI: 0.10–0.20) showed significant heterogeneity (*I^2^* = 98.4; *p* < 0.001). The subgroup analysis revealed that the most affected host was *Sarotherodon,* with a prevalence of 0.42 (95% CI: 0.22–0.65). Cestode was the taxonomic group with the largest prevalence (0.40; 95% CI:0.32–0.48), followed by amoeba (0.24; 95% CI: 0.16–0.35) and nematode (0.22; 95% CI: 0.11–0.38), among which, *Schyzocotyle* spp., *Opistorchis* spp., *Gnathostoma* spp. and *Vermamoeba* spp. have an impact on public health. Significant differences (*p* < 0.004) were found among continents and countries, with the highest value of prevalence detected in the African continent (0.28; 95% CI: 0.20–0.37), specifically in Tanzania (0.56; 95% CI: 0.22–0.87) and Egypt (0.43; 95% CI: 0.20–0.55). The origin of samples had a significant effect (*p* < 0.0001) on the detected prevalence, especially from those that showed the highest prevalence (0.24; 95% CI: 0.17–0.33). Finally, there were no differences in prevalence according to the diagnostic test (*p* = 0.97). Our results provide useful information on the development of epidemiological programs for the control of zoonoses associated with parasites in tilapia and in the design, planning, and implementation of future research.

## 1. Introduction

Aquaculture is one of the most important food production sectors worldwide, as it has been growing steadily over the last few decades, contributing to the achievement of many of the Sustainable Development Goals established by the United Nations, including, among others, poverty reduction, nutrition, sustainability, good health and wellbeing, and the generation of sources of employment [1]. In 2018, worldwide aquaculture production reached 114.5 million tons; and within this, “tilapia” species contributed 6 million tons [2,3].

Tilapia species (family *Cichlidae*) are African fish from tropical environments. There are three genera of economic importance in aquaculture, *Oreochromis*, *Sarotherodon,* and *Tilapia* [4]. In general, tilapia is characterized by rapid growth and its ability to colonize various aquatic environments. They are very resistant to low oxygen levels (below 4 mg O_2_/L) and high concentration of organic matter in the water (more than 30 mg of suspended solids) [5]. Thus, tilapia are able to survive in high variations of salinity and temperature [6]. These characteristics, together with its relatively easy reproduction, have made tilapia one of the most cultivated species in the world, and it is expected that its production will continue to grow to reach 7.3 million tons by 2030 [7,8].

However, as in any other farming industry, tilapia is not exempt from diseases associated with this wide diversity of etiological agents, and this could include zoonotic parasites [9,10]. A zoonosis is an infectious disease that can be transmitted from animals to humans [11]. In most cases, zoonotic parasitic diseases in human beings from fish are accidental infections [12,13] caused by ingestion of undercooked meat containing viable parasites [14].

Among zoonotic diseases, there is a wide diversity of etiological agents that cause them, such as protozoa, worms, and arthropods, many of them have important implications for public and veterinary health. For instance, estimates indicate that 500 million people are at parasitosis risk in the world [15]. Indeed, there are epidemiological reports estimating that 18 million people in Asia are infected with trematodes, while in developing areas, such as Africa, up to 81 million people are at risk of helminth infections [16,17,18]. Within the causative agents of these infections, some of those that are transmitted by the consumption of tilapia have been identified as trematodes, such as *Haplorchis pumilio*, *Centrocestus formosanus*, *Haplorchis yokogawi*, *Pygidiopsis genata,* and *Phagicola ascolonga* [16,19], nematodes, such as *Gnathostoma* spp., emergent zoonotic parasites, and *Contracaecum* spp. [12,20,21,22]. In addition, there are reports of cestodes in tilapias (*Schyzocotyle* spp.) and protozoan infections with zoonotic potential, such as *Giardia duodenalis* [23], *Cryptosporydium parvum* [24,25], and *Vermamoeba vermiformis* [26]. In free-living protozoan infections, tilapia can function as a vector for the transmission of the infection that can even occur by contact [27].

Zoonotic parasites can lead to particular signs of disease in fish, including skin hemorrhagic lesions [28], laminar melting in gills [29,30], intestinal disorders [31], and finally, mortality. In human beings, pathology goes from subclinical symptoms to abdominal pain, vomiting, fever, and weight loss [23,25]; in severe clinical cases, there is the presence of eosinophilic granuloma in the heart, brain, and spine due to the nematode infection, such as the outbreak of gnathostomiasis reported in Mexico in the 1970s, where the origin is attributed to tilapia consumption from the Miguel Aleman dam. Indeed, cholangiocarcinoma has been observed in trematode infection [13,14,19,32,33,34]. Given this scenario, the aim of this work is to assess the zoonotic parasite prevalence associated with the ingestion of tilapia meat. In addition, it is important to address the occurrence and distribution aspects, which can help develop intervention strategies in public health and parasite control.

## 2. Materials and Methods

### 2.1. Data Sources and Searches

A systematic literature search via ScienceDirect (https://www.sciencedirect.com/, accessed on 8 August 2022), PubMed (http://www.pubmed.gov, accessed on 8 August 2022), PRIMO-UAEH (https://uaeh-primo.hosted.exlibrisgroup.com/primo-explore/search?vid=UAEH, accessed on 8 August 2022), CONRICyT (https://www.conricyt.mx/, accessed on 8 August 2022), LILACS (https://lilacs.bvsalud.org/es/, accessed on 8 August 2022), and AJOL (https://www.ajol.info/index.php/ajol, accessed on 8 August 2022) was carried out using a predetermined protocol in accordance with the preferred reporting items for systematic reviews and meta-analysis (PRISMA) in order to identify scientific publication that report prevalence of zoonotic parasite diseases in tilapia, search engines were reviewed during the months of August to November 2020. LILACS and AJOL are databases specialized in Latin American and African scientific literature in health sciences, respectively. Searches were limited to articles in English. The zoonotic potential of parasites that affect tilapia was defined by the available scientific literature, as is shown in Table S1.

The search was conducted in three stages. First, we carried out a general search of studies focused on parasites that affect tilapia using the following keywords: “parasite”, “nematodes”, “cestodes”, “trematodes”, “cichlids”, “*Oreochromis*” and “tilapia”. The second stage comprised the search of studies focused on parasites that affect tilapia with zoonotic potential using the following terms: “zoonotic parasite”, “foodborne parasites”, “Oreochromis” and “tilapia”. Finally, the third stage focused on searching for prevalence studies using specific keywords of parasite genera and diseases in combination with research terms to refer to host organisms (“*Oreochromis*” and “tilapia”). These entries included the main helminths reported in tilapia (nematodes, cestodes, and trematodes). The full approach and keywords used are depicted in Table 1.

### 2.2. Study Selection and Eligibility Criteria

The search was performed by three field experts to avoid reviewer bias. Two study authors independently screened the search output to identify full texts that met the following eligibility criteria: (a) studies published in an international peer-reviewed scientific journal, (b) studies that reports prevalence of zoonotic parasite diseases in tilapia, even if prevalence was 0%, (c) articles that reported the number of events per study, and (d) articles that reported the study population. An event was defined as a positive diagnosis of zoonotic parasites that affect tilapia. In the current study, “tilapia” fish correspond to cichlids of the genera *Oreochromis* spp., *Sarotherodon* spp., *Ptychochromis* spp., *Vieja* spp., and *Tilapia* spp.; also, native, or wild cichlids that maintain the name “tilapia” were included. Studies that assessed zoonotic parasite diseases in water, soil, and environmental samples were omitted, since they did not provide prevalence data.

### 2.3. Data Extraction and Tabulation

After selecting the studies that met the previous eligibility criteria, one expert extracted data into a spreadsheet and the other two reviewers verified the database for any discrepancy. Conflicts were resolved by consensus or by consulting a third researcher. The final database included 52 articles, from which the following information was obtained: article title, authors’ name, journal of publication, and publication year. Interest variables were categorized as primary outcomes and exploratory outcomes. The primary outcomes correspond to quantitative response variables (i.e., prevalence), while the exploratory outcomes describe the environmental and experimental characteristics of the analyzed studies. Both types of outcomes were obtained according to the available information in the analyzed scientific articles (Table 2).

### 2.4. Meta-Analysis

A meta-analysis was carried out to estimate the global prevalence of zoonotic parasites that affect tilapia. For estimation of prevalence, we extracted data from the number of events and the total number of samples to perform proportional meta-analysis in the R environment for statistical computing (version 4.0.2; R Core Team, 2020) using the “metaprop” function of ‘meta’ package version 4.13–0 [35]. Polled proportions of prevalence were estimated using a logit transformation in a logistic-normal random-effect regression model as described by Nyaga et al. (2014) [36].

(1) $$r_{i}\sim binomial\;\left(p_{i},\;n_{i}\right)$$

where observed events *r_i_* assumed a binomial distribution with parameters *p_i_* and sample size *n_i_*. Therefore, a normal distribution was used for the random-effects model.

(2) $$logits\left(p_{i}\right)\sim normal\left(\mu ,\tau \right)$$

where μ is the mean of a population of possible means and τ  is the between-study variance, both on the logit scale. The exact or Clopper-Pearson method [37] to binomial proportions was used to construct confidence intervals for individual studies.

Since the analyzed studies were conducted across different environmental conditions, procedures of sample collection and diagnostic methods, the prevalence of zoonotic parasites was expected to show high heterogeneity. Heterogeneity was explored by inspection of a forest plot, estimation of between-study random-effects variance (*t*^2^) and the percentage of variability explained by heterogeneity rather than by a simple variance (*I*^2^ index) [38]. The between-study variance (*t*^2^) was estimated using the maximum-likelihood estimator method. We investigated sources of heterogeneity between studies through subgroup analysis with respect to continent and country from which the study was conducted, genus of the host, parasite taxonomic group, sample origin, and type of diagnostic test. The results are presented as summary proportions with 95% confidence intervals and were displayed in a forest plot as well as heterogeneity between subgroups.

## 3. Results

**Figure 1:**
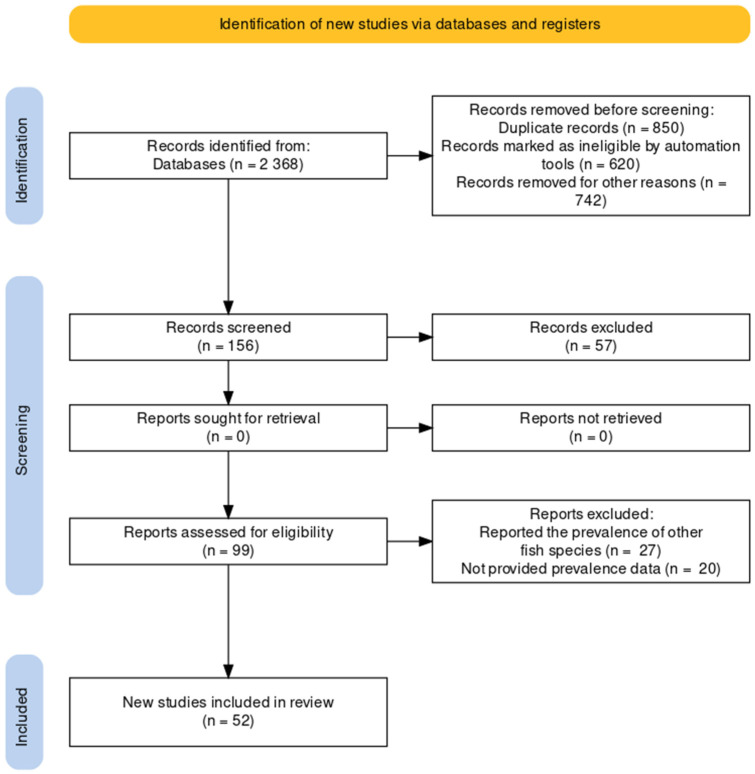
Flow diagram describing the paper selection process according to PRISMA guidelines.

The flow diagram of search results is displayed in Figure 1. The systematic search through electronic databases yielded a total of 1044 articles. First, the screening stage excluded 724 studies, mainly due to replication and being outside of the scope of the current study. In the second stage, 171 duplicated studies were removed; to the rest of the studies (*n* = 149), eligibility criteria were applied; in this stage, the main reasons for exclusion were: reports of zoonotic parasitic diseases of non-tilapia fish and studies that do not report prevalence. Fifty-two articles containing prevalence data of diseases caused by zoonotic parasites in tilapia met all criteria for inclusion and were used for meta-analysis (Figure 1).

A total of 19,247 observations (sample size) were analyzed in order to identify the global prevalence of parasitic infections with zoonotic potential in tilapia, with a pooled prevalence of 0.14 (95% CI: 0.10–0.10) showing a significant heterogeneity (*I*^2^ = 98.4; *p* < 0.001).

In the current meta-analysis, heterogeneity was explored through a subgroup analysis. Most of the subgroup analyses of exploratory outcomes showed considerable heterogeneity (*I*^2^ > 86.0). Significant statistical heterogeneity in the subgroup analysis reveals a likely interaction among exploratory variables. Only the protozoan taxonomic group displayed low heterogeneity (*I*^2^ = 0). Heterogeneity was not possible to calculate for variables with only one study per group (host: *Ptychochromis*, *Vieja*; taxonomic group: amoeba, cestode; sample origin: restaurants). However, these studies were not eliminated because they contributed to the estimation of the overall prevalence.

The subgroup analysis with respect to the host resulted in a significant difference within the subgroup (*p* < 0.0001). The parasitic prevalence in tilapia assessed by the host ranges from a minimum overall prevalence of 0.09 (95% CI: 0.06–0.15) to the *Oreochromis* group with the highest number of outcome observations (*n* = 14,379). The *Tilapia* group shows a meta-analysis overall prevalence of 0.19 (95% CI: 0.10–0.34) with the second largest number of observations (*n* = 3923). The largest values of prevalence were shown by *Ptychochromis* (0.40; 95% CI: 0.32–0.48) and *Sarotherodon* (0.42; 95% CI 0.22–0.65); however, these results must be taken with caution due to the low sample size used to estimate the overall prevalence (Table 3).

**Figure 2:**
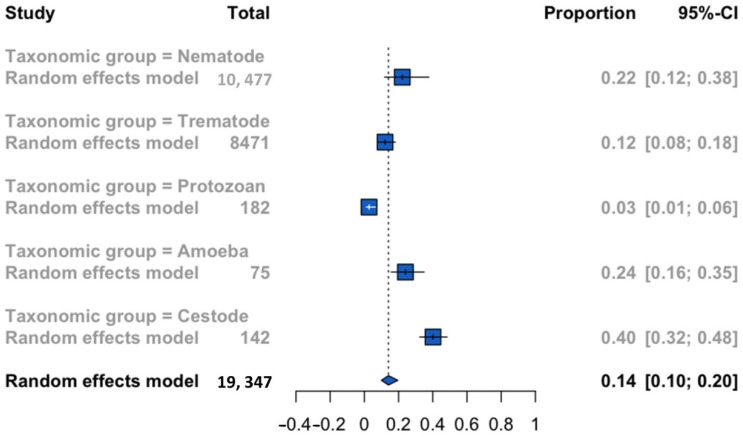
Forest plot of the global prevalence of parasitic infections with zoonotic potential in tilapia according to the taxonomic group.

Subgroup analysis based on the taxonomic group revealed a significant effect of this moderator outcome (*p* < 0.0001). Five parasite taxonomic groups were identified that affected tilapia populations. The cestode taxonomic group showed the highest overall prevalence (0.40; 95% CI: 0.32–0.48), followed by amoeba infections (0.24; 95% CI: 0.16–0.35); however, these results should be interpreted cautiously due to the low number of analyzed studies that do not allow heterogeneity estimation. Taxonomic groups with the highest sample size were trematode (*n* = 8471) and nematode (10,477) with a pooled prevalence of 0.12 (95% CI: 0.08–0.18) and 0.22 (95% CI: 0.12–0.38), respectively. The lowest prevalence of parasitic infection in tilapia was shown by the protozoan taxonomic group (0.03; 95% CI: 0.01–0.06) (Figure 2).

**Figure 3:**
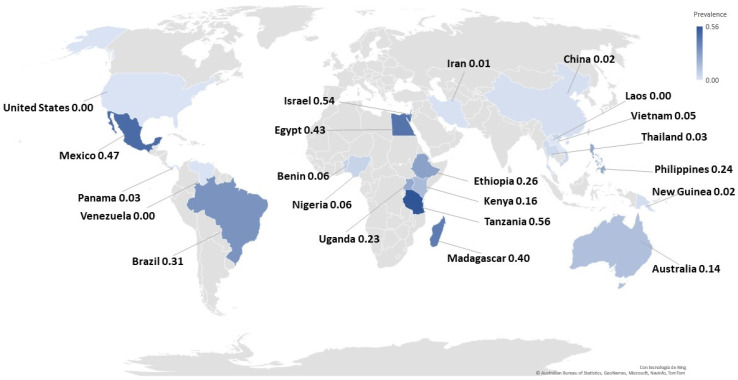
Geographical distribution of global prevalence of parasitic infections with zoonotic potential in tilapia.

According to moderator analysis, prevalence shows considerable geographical variation (continent and country) (Table 3). Regarding the continent, America had the largest sample size (*n* = 8409) with a prevalence of 0.13 (95% CI: 0.00–0.41) and Mexico had the largest number of observations (*n* = 8280). The African continent had the highest positive rate of 0.28 (95% CI: 0.20–0.37) and the largest number of countries reporting parasitic infections. Tanzania (0.56; 95% CI: 0.22–0.87) and Egypt (0.43; 95% CI: 0.20–0.55) were the countries with the largest values of prevalence. Conversely, Oceania showed the lowest prevalence (0.07; 95% CI: 0.00–0.22) because Australia is the only country that reports parasitic infections in tilapia (0.14; 95% CI: 0.15–0.34) (Figure 3).

The analysis results showed a variation in global prevalence based on the origin of samples (*p* < 0.0001). Subgrouping analysis revealed that most studies were carried out on fishing samples (*n* = 15,615) with a global prevalence of 0.24 (95% CI: 0.17–0.33). The prevalence of aquaculture studies was 0.05 (95% CI: 0.02–0.11) with the second largest number of observations (*n* = 2872). The prevalence of parasites from restaurant samples was obtained from a single study. Sixty-five observations were examined in this study, of which the prevalence estimated was 0.15 (95% CI: 0.08–0.26) (Table 3). There were no differences in the prevalence according to the diagnostic test (*p* = 0.97). Parasitic prevalence was 0.14 (95% CI: 0.09–0.20) and 0.13 (95% CI: 0.07–0.24) for microscopy and PCR, respectively (Table 3).

**Figure 4:**
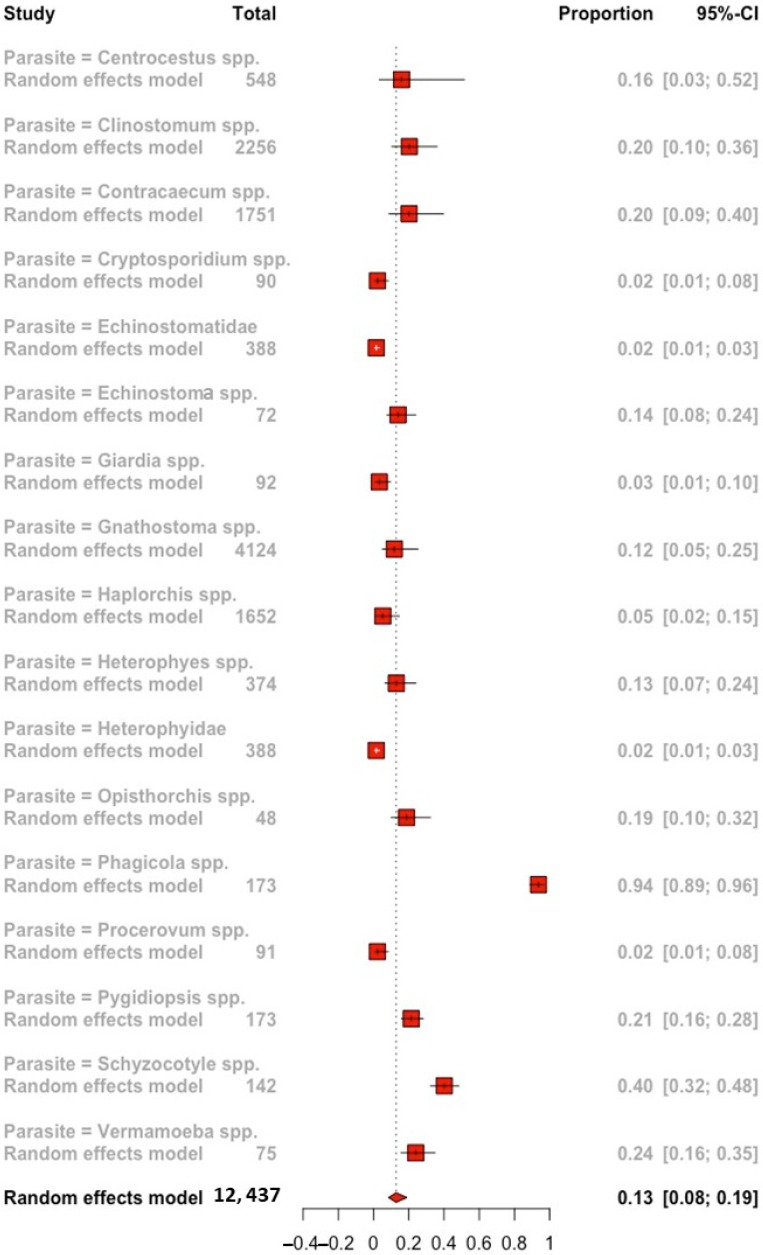
Forest plot of the global prevalence of parasitic infections with zoonotic potential in tilapia according to parasitic genera and family according to the available information.

The parasitic genera that were identified in the tilapia samples were 15. In addition, two positive samples were identified at the family level (Echinostomatidae and Heterophydae); the mean effect calculated for the explanatory variable “Parasite” was 0.13 (95% CI: 0.08–0.19) with a 98% *I*^2^. Parasites of the genus *Gnathostoma* spp. (4124), *Clinostomum* spp. (2256), and *Contracaecum* spp. (1751) showed the highest sample size in the random-effects model, while it was *Phagicola* spp. and *Schyzocotyle* spp. that showed a higher prevalence rate of 0.94 (95% CI: 0.89–0.96) and 0.40 (95% CI: 0.32–0.48), respectively. Moreover, the genus *Cryptosporidium* spp. had the lowest clustered prevalence rate (0.02; 95% CI: 0.01–0.08) (Figure 4).

## 4. Discussion

This systematic review and meta-analysis showed an overall prevalence of 0.14 (95% CI: 0.10–0.20) for zoonotic parasites in tilapia and a lower prevalence for pathological infections in tilapia by protozoan parasites (69%). However, our prevalence was higher than crustacean infections (5%) [39]. The variability in overall prevalence can be explained by the wide diversity of etiologic agents reported; fish were documented to be affected by up to 15 parasitic genera of trematodes (9), cestodes (1), nematodes (2), and protozoa (3). The general heterogeneity calculated showed an interaction between the bibliographic resources selected for the meta-analysis (Table 3). This characteristic allowed for directing the use of a random-effects model to generate the estimate of the pooled prevalence with 95% confidence intervals [40].

According to evaluated data, five tilapia genera were identified with zoonotic parasite infection: *Oreochromis* spp., *Tilapia* spp., *Sarotherodon* spp., *Ptychochromis* spp., and *Vieja* spp. The first two were shown as important zoonotic parasitic load reservoirs. The high observation number in *Oreochromis* spp. (*n* = 14,379) evidenced it as the *Tilapia* genus with the highest recurrence in cultivation practices and repopulation in bodies of continental water worldwide. In addition, it has the highest number of species described in any of the genus, with a value of 32 [4,41]. Jointly, the genus *Tilapia* presented a high sample size (*n* = 3923), likely related to recurrent production and consumption on the African continent [42]. These genera have exponents, such as *Oreochromis niloticus* and *Tilapia zillii*, as excellent crop species due to their tolerance to temperature, oxygen and salinity, rapid growth, and high environmental adaptability, which has allowed their introduction to different countries [43]. Moreover, a higher prevalence rate was observed in *Sarotherodon* spp. and *Ptychochromis* spp., fishes with a slower growth rate worldwide; for example, *S. melanotheron* fish is still in the domestication process for production [44], while various species, such as *Ptychochromis* spp., have high endemism in Madagascar, Africa, which makes them difficult to disperse and cultivate for use [45]. The evaluation suggests that captured species and recent domestication attempts influence the prevalence of zoonotic infections by parasites.

In our study, the parasites determined in tilapia samples showed a higher prevalence of infections caused by cestodes (0.40; 95% CI: 32–48), which could be partially explained by the low-host specificity of these parasites [46]. However, these results should be taken with caution because cestodes had a low sample size to estimate the global prevalence (*n* = 142). Life-cycle characteristics may influence the low number of cestodes in tilapia because these parasites require copepods (*Acanthocyclops* spp. and *Cyclops* spp.) as primary hosts, which conditions a higher infection frequency in planktonic diet fish, such as cyprinids [47].

The wide presence of nematode infection in tilapia can be related to its wide biological diversity, since approximately 25,000 species have been recognized for the entire phylum [48]. Additionally, the higher number of observed cases of nematode infections can be associated with the increase in Gnathostomiasis cases in humans. Actually, nematode infection has been classified as an emerging disease in countries, such as Mexico, Guatemala, Colombia, Ecuador, Peru, and Brazil, which represents a public health problem related to tilapia consumption [49,50,51,52]. It has also been reported that infections by the nematode *Contracaecum* spp. in humans have been associated with vomiting, diarrhea, and abdominal syndrome [53]. According to our findings, trematodes showed a moderate prevalence with a high sample size and a wide diversity of parasites that affect tilapia. The infection potential of trematodes is probably associated with their life-cycle characteristics due to these parasites having motility stages (miracide and cercariae) that facilitate the search for their hosts, conferring an advantage in establishing a parasite-host relationship [54]. Among zoonotic trematodes that infect fish, we found that the Heterophyidae and Echinostomatidae families, which are associated with intestinal infection, and the Opisthorchiidae family, provoke liver infection in humans [17]. In relation to the presence of protozoa, these have been related to intestinal infections in humans, which reflect signs of abdominal pain [26]. Also, in this study, *Cryptosporidium* spp. and *Giardia* spp. showed low prevalence in the analyzed tilapias, while *Vermamoeba* spp. presented higher values (0.24; 95% CI: 0.16–0.35).

Geographical distribution in data assessment showed prevalence in 22 countries (Figure 3), where Africa (eight countries) showed the highest clustered prevalence rate (0.28; 95% CI: 0.20–0.37). Egypt and other sub-Saharan African countries maintain high tilapia production [55]; unfortunately, many African countries face constant challenges, such as lack of safe drinking water, inadequate sanitation, and hygiene, as well as diagnosis and treatment of disease difficulties [56]. These factors could enhance the dispersion and transmission of zoonotic parasites in the consumption of tilapia and other fish species. Asia has extensive tilapia production capacity in countries, such as China [57,58], which also depicts a high endemism of zoonotic parasites in the south-east of the continent [59]. According to our results, Asia presented a low clustered prevalence rate (0.10; 95% CI: 0.04–0.18), which shows a lack of attention to public health problems by some countries in the area. The United States Agency for International Development (USAID) has participated through mass medication against tropical diseases on the Asian continent [60]. In addition, water, hygiene, and sanitation programs have been implemented [61].

On the other hand, America accounts for the highest sample size (*n* = 8409), with Mexico as a country of endemism for *Gnathostoma* spp. [51,52,62,63], which has caused the monitoring of the etiologic agent causing Gnatostomiasis [20] and the inclusion of *Gnathostoma* spp. in the trade policies of this country [64]. However, zoonotic parasitic distribution has high potential due to various factors: host migration [65], culinary habit globalization [66,67,68], market policy omission [53,69], parasite-fish cointroduction [41,46], and even migration and tourism [59,70].

Our study showed the greatest prevalence in wild fish (0.24; 95% CI: 0.17–0.33), evidencing immunological differences, resistance, and tolerance to parasitic loads between wild and crop populations [71]. The lower prevalence rate in controlled environments (culture) can be related to quarantine application, rejection of batches, personnel control, and quality of food supplied, among others [72], while in wild environments, biotic factors are combined without restrictions that allow intermediate hosts and perpetuate the life cycle of parasites [73,74]. Likewise, in wild environments, pollution factors may favor the growth of the population of parasites [75,76]. The diagnostic test explanatory variable did not show significant statistical differences (*p* = 0.97) in the grouped prevalence. This shows the use of molecular techniques as a complement. In addition, the scope of this analysis was at the level of parasitic genera, for which future studies are suggested for evaluation at the species level.

Among parasitic genera, the highest grouped prevalence rates in tilapia populations were cestode *Schyzocotyle* spp. (0.40; 95% CI: 0.32–0.48). These parasites usually infect a wide fish variety, however, in humans, there are reports of accidental infection by egg stage, where signology is manifested with abdominal pain [77]. Our study suggests that emerging infections affect tilapia populations largely because the fish have not been previously exposed to these pathogens, so they lack regulation in the expression of genes related to the adaptive immune system necessary to combat these infections [63,78]. Moreover, infections by reoffending genera showed lower prevalence, reflecting host-parasite relationship coevolution, where both have been exposed to antagonistic natural selection processes, allowing the ecological relationship continuity [79]. *Clinostomum* spp. is an example. This parasite presented 2256 observations and has the lowest pooled prevalence rates (0.20; 95% CI: 0.10–0.36) because this etiological agent has been described with high specificity of infection in tilapia [80]. The forest plot of the explanatory variable “Parasite” shows this behavior (Figure 4).

Finally, nine genera were evaluated corresponding to trematoda taxon; *Centrocestus* spp., *Clinostomum* spp., *Echinostoma* spp., *Haplorchis* spp., *Heterophyes* spp., *Opisthorchis* spp., *Phagicola* spp., *Procerovum* spp., and *Pygidiopsis* spp. Trematode infections in tilapia populations can be promoted by the fact that these parasites use gastropod molluscs as primary hosts. These hosts are persistent in aquatic environments and widely distributed worldwide, such as in the case of *Melanoides tuberculata* and *Pirenella conica* [59,81,82], a situation that makes it difficult to control parasitic loads. In view of the aforementioned, this evaluation aims to provide a basic guide for continuing the establishment of monitoring programs and the development of intervention strategies, against zoonotic parasitic loads in the tilapia production and consumption chain. Similarly, this information can promote compliance with public policies in countries that have applicable regulations for tilapia meat safety. In Mexico, the regulations indicate the use of refrigerated and frozen aquaculture products, likewise, the correct cooking before consumption of fish meat, including tilapia meat, is indicated [64]

## 5. Conclusions

The results of the meta-analysis reported a global prevalence of 0.14 (95% CI: 0.09–0.19) of analyzed studies. The parasites *Phagicola* spp. and *Schyzocotyle* spp. presented the highest prevalence in the study. The variability of the global effect of the prevalence of parasites in tilapia was partially associated with the explanatory variables. For instance, the fishing reports and samples of the genus *Sarotherodon* spp. presented the highest values of prevalence. Except for the independent variable “sample type”, most of the analyzed groups displayed a heterogeneity greater than 90%. The prevalence of parasites in tilapia showed variation according to the geographical region where the study was carried out, where Africa presented a greater number of studies with prevalence for zoonotic parasites; this variability could be associated with experimental characteristics, socioeconomic factors, and culinary practices. According to the results, the level of prevalence depends on the characteristics of the experimental design, such as sample type, sample size, and diagnostic technique. Therefore, the assessments of explanatory variables provide useful information in the design, planning, and implementation of future research with the optimization of materials and human resources. Additionally, they also provide information about the design and development of epidemiological programs for the control of zoonoses associated with parasites in tilapia. They join the preventive measures and good consumer practices available.

## Figures and Tables

**Table 1:** Search strategy for zoonotic parasites studies that affect tilapia.

1	(Oreochromis or Tilapia ^1^ or Cichlids) and (Parasite or Nematode or Cestode or Trematode)
2	zoonotic parasite/or foodborne parasites/or *Oreochromis*/or Tilapia ^1^/
3	prevalence/or *Diphyllobothrium*/or Diphyllobothriasis/or *Oreochromis*/or Tilapia ^1^/
4	prevalence/or *Bothriocephalus* or *Schyzocotyle*/or Bothriocephaliasis/or *Oreochromis*/or Tilapia ^1^/
5	prevalence/or *Centrocestus*/or Centrocestiasis/or *Oreochromis*/or Tilapia ^1^/
6	prevalence/or *Clonorchis*/or Clonorchiasis/or *Oreochromis*/or Tilapia ^1^/
7	prevalence/or *Opisthorchis*/or Opisthorchiasis/or *Oreochromis*/or Tilapia ^1^/
8	prevalence/or *Heterophyes*/or Heterofiasis/or *Oreochromis*/or Tilapia ^1^/
9	prevalence/or *Haplorchis*/or Haplorchiasis/or Oreochromis/or Tilapia ^1^/
10	prevalence/or *Gnathostoma*/or Gnathostomiasis/or *Oreochromis*/or Tilapia ^1^/

^1^ Word “Tilapia” used in the search refers to the cichlid fish group commonly named tilapia.

**Table 2:** Data variables extracted from studies included in the meta-analysis.

Primary Outcomes
Number of events per study (positive cases)
Size of study population
Prevalence
**Exploratory outcomes**
Host fish (genera)
Parasite (genera and species)
Parasite taxonomic group (nematode, trematode, protozoan, amoeba independently tested for their free-living in ponds, and cestode)
Sample origin (aquaculture, fishing, restaurants, and aquaculture-fishing)
Diagnostic test (microscopy and PCR)
Country of study
Continent of study

**Table 3:** Results of meta-analysis assessing the prevalence of parasitic infections with zoonotic potential in tilapia.

Item	Summary Proportion	Lower 95% CI	Upper 95% CI	*I* ^2^	Sample Size
**Overall estimated**	0.14	0.10	0.20	98.4	19,347
**Host**					
*Oreochromis*	0.09	0.06	0.15	99.0	14,379
*Tilapia*	0.19	0.10	0.34	98.0	3923
*Sarotherodon*	0.42	0.22	0.65	98.0	863
*Ptychochromis*	0.40	0.32	0.48	n.e.	142
*Vieja*	0.02	0.003	0.15	n.e.	40
p-value *	<0.0001				
**Taxonomic group**					
Amoeba	0.24	0.16	0.35	n.e.	75
Cestode	0.40	0.32	0.48	n.e.	142
Nematode	0.22	0.11	0.38	99.4	10,477
Protozoan	0.03	0.01	0.06	0.0	182
Trematode	0.12	0.08	0.18	96.3	8471
p-value *	<0.0001				
**Continent**					
Africa	0.28	0.20	0.37	98.1	6420
America	0.13	0.00	0.41	99.8	8409
Asia	0.10	0.04	0.18	99.0	4356
Oceania	0.07	0.00	0.22	97.5	162
p-value *	<0.0004				
**Sample origin**					
Aquaculture	0.05	0.02	0.11	92.6	2872
Fishing	0.24	0.17	0.33	98.8	15,615
Restaurants	0.15	0.08	0.26	n.e.	65
Aquaculture-fishing	0.05	0.03	0.11	86.0	795
p-value *	<0.0001				
**Diagnostic test**					
Microscopy	0.14	0.09	0.20	98.2	14,476
PCR	0.13	0.07	0.24	96.6	4871
p-value *	0.97				

* *p*-value of test for subgroup differences of the random-effects model; n.e., not estimable.

## Data Availability

Not applicable.

## Supplementary Materials

The following are available online at https://www.mdpi.com/article/10.3390/ani12202800/s1, Table S1: Parasites of tilapia with zoonotic potential according with the available scientific literature [83,84,85,86,87,88,89,90,91,92,93,94,95,96,97,98,99,100,101,102,103,104,105,106,107,108,109,110,111,112,113,114,115,116,117].

## References

[1] Béné C., Arthur R., Norbury H., Allison E.H., Beveridge M., Bush S., Campling L., Leschen W., Little D., Squires D. (2016). Contribution of Fisheries and Aquaculture to Food Security and Poverty Reduction: Assessing the Current Evidence. World Dev..

[2] Food Agriculture Organization (FAO) (2020). El estado mundial de la Pesca y la Acuicultura. Mar. Pollut. Bull..

[3] Savaya A., Glassner H., Livne-Luzon S., Chishinski R., Molcho J., Aflalo E.D., Zilberg D., Sagi A. (2020). Prawn monosex populations as biocontrol agents for snail vectors of fish parasites. Aquaculture.

[4] El-Sayed A.-F.M., El-Sayed A.-F.M. (2020). Taxonomy and Basic Biology. Tilapia Culture.

[5] Arguedas D., Ortega C., Martínez S., Astroza A. (2017). Parasites of Nile Tilapia larvae *Oreochromis niloticus* (Pisces: *Cichlidae*) in concrete ponds in Guanacaste, Northern Costa Rica. UNED Res. J..

[6] Fajer-Ávila E.J., Medina-Guerrero R.M., Morales-Serna F.N. (2017). Strategies for prevention and control of parasite diseases in cultured tilapia. Acta Agrícola Pecu..

[7] Abdel-Latif H.M.R., Dawood M.A.O., Menanteau-Ledouble S., El-Matbouli M. (2020). The nature and consequences of co-infections in tilapia: A review. J. Fish Dis..

[8] Vega F., Cortés M., Zuñiga L., Jaime B., Galindo J., Basto M. (2010). Small-scale culture of tilapia (Oreochromis niloticus), alimentary alternative for rural and peri-urban families in Mexico?. Redvet.

[9] Bao M., Pierce G.J., Strachan N.J.C., Pascual S., González-Muñoz M., Levsen A. (2019). Human health, legislative and socioeconomic issues caused by the fish-borne zoonotic parasite Anisakis: Challenges in risk assessment. Trends Food Sci. Technol..

[10] Carrique-Mas J.J., Bryant J.E. (2013). A review of foodborne bacterial and parasitic zoonoses in Vietnam. Ecohealth.

[11] Organización Mundial de la Salud (OMS) Zoonosis. 2022. p 1. https://www.who.int/es/news-room/fact-sheets/detail/zoonoses.

[12] Chibwana F.D., Mshana J.G., Katandukila J.V. (2020). A Survey of Fish Parasites from Pangani Catchment and Lake Kitangiri in Singida, Tanzania. Tanzania J. Sci..

[13] Hung N.M., Dung D.T., Anh N.T.L., Van P.T., Thanh B.N., Van Ha N., Van Hien H., Canh L.X. (2015). Current status of fish-borne zoonotic trematode infections in Gia Vien district, Ninh Binh province, Vietnam. Parasit Vectors.

[14] Wang D., Young N., Korhonen P., Gasser R. (2018). Clonorchis sinensis and Clonorchiasis: The Relevance of Exploring Genetic Variation. Advances in Parasitology.

[15] Chi T.T., Dalsgaard A., Turnbull J., Tuan P., Murrell D. (2008). Prevalence of zoonotic trematodes in fish from a vietnamese fish-farming community. J. Parasitol..

[16] Ibrahim M.M., Soliman M.F.M. (2010). Prevalence and site preferences of heterophyid metacercariae in *Tilapia zilli* from Ismalia fresh water canal. Egypt. Parasite.

[17] Wiriya B., Clausen J.H., Inpankaew T., Thaenkham U., Jittapalapong S., Satapornvanit K., Dalsgaard A. (2013). Fish-borne trematodes in cultured Nile tilapia (*Oreochromis niloticus*) and wild-caught fish from Thailand. Vet. Parasitol..

[18] Mardu F., Yohannes M., Tadesse D. (2019). Prevalence of intestinal parasites and associated risk factors among inmates of Mekelle prison, Tigrai Region, Northern Ethiopia. BMC Infect. Dis..

[19] Hop N.T., De N.V., Murrell D., Dalsgaard A. (2007). Occurrence and species distribution of fishborne zoonotic trematodes in wastewater-fed aquaculture in northern Vietnam. Trop. Med. Int. Heal..

[20] Diaz Camacho S.P., Willms K., Ramos M., Del Carmen de la Cruz Otero M., Nawa Y., Akahane H. (2002). Morphology of *Gnathostoma* spp. isolated from natural hosts in Sinaloa, Mexico. Parasitol. Res..

[21] Motamedi M., Iranmanesh A., Teimori A., Sadjjadi S.M., Nasibi S. (2019). Detection of *Contracaecum multipapillatum* (Nematoda: *Anisakidae*) in the indigenous killifish *Aphanius hormuzensis* (Teleostei; *Aphaniidae*) and its histopathological effects: A review of Iranian Aphanius species parasites. J. Appl. Ichthyol..

[22] Salgado-Maldonado G., Aguilar-Aguilar R., Cabañas-Carranza G., Soto-Galera E., Mendoza-Palmero C. (2005). Helminth parasites in freshwater fish from the Papaloapan river basin, Mexico. Parasitol. Res..

[23] Ghoneim N.H., Abdel-Moein K.A., Saeed H. (2012). Fish as a possible reservoir for zoonotic *Giardia duodenalis* assemblages. Parasitol. Res..

[24] Couso-Pérez S., Ares-Mazás E., Gómez-Couso H. (2018). Identification of a novel piscine *Cryptosporidium* genotype and *Cryptosporidium parvum* in cultured rainbow trout (*Oncorhynchus mykiss*). Parasitol. Res..

[25] Koinari M., Karl S., Ng-Hublin J., Lymbery A.J., Ryan U.M. (2013). Identification of novel and zoonotic *Cryptosporidium* species in fish from Papua New Guinea. Vet. Parasitol..

[26] Milanez G.D., Masangkay F.R., Thomas R.C., Ordona M.O.G.O., Bernales G.Q., Corpuz V.C.M., Fortes H.S.V., Garcia C.M.S., Nicolas L.C., Nissapatorn V. (2017). Molecular identification of *Vermamoeba vermiformis* from freshwater fish in lake Taal, Philippines. Exp. Parasitol..

[27] Chelkha N., Hasni I., Louazani A.C., Levasseur A., La Scola B., Colson P. (2020). *Vermamoeba vermiformis* CDC-19 draft genome sequence reveals considerable gene trafficking including with candidate phyla radiation and giant viruses. Sci. Rep..

[28] Echi P., Okafor F., Eyo J. (2009). Co-infection and morphometrics of three clinostomatids (Digenea: *Clinostomatidae*) in *Tilapia guinensis* Bleeker, 1862 from Opi lake, Nigeria. Bio Res..

[29] Fleming P.B., Huffman D.G., Bonner T.H., Brandt T.M. (2011). Metacercarial distribution of *Centrocestus formosanus* among fish hosts in the Guadalupe river drainage of Texas. J. Aquat. Anim. Health.

[30] Mahmoud M.A., Abdelsalam M., Mahdy O., El Miniawy H.M., Ahmed Z.A., Osman A.H., Mohamed H., Khattab A., Ewiss M.Z. (2016). Infectious bacterial pathogens, parasites and pathological correlations of sewage pollution as an important threat to farmed fishes in Egypt. Environ. Pollut..

[31] Morine M., Yang R., Ng J., Kueh S., Lymbery A.J., Ryan U.M. (2012). Additional novel *Cryptosporidium* genotypes in ornamental fishes. Vet. Parasitol..

[32] Dao H.T.T., Dermauw V., Gabriël S., Suwannatrai A., Tesana S., Nguyen G.T.T., Dorny P. (2017). *Opisthorchis viverrini* infection in the snail and fish intermediate hosts in Central Vietnam. Acta Trop..

[33] Elsheikha H.M., Elshazly A.M. (2008). Host-dependent variations in the seasonal prevalence and intensity of heterophyid encysted metacercariae (Digenea: *Heterophyidea*) in brackish water fish in Egypt. Vet. Parasitol..

[34] Elsheikha H.M., Elshazly A.M. (2008). Preliminary observations on infection of brackish and fresh water fish by heterophyid encysted metacercariae in Egypt. Parasitol. Res..

[35] Balduzzi S., Rücker G., Schwarzer G. (2019). How to perform a meta-analysis with R: A practical tutorial. Evid. Based Ment. Health.

[36] Nyaga V.N., Arbyn M., Aerts M. (2014). Metaprop: A Stata command to perform meta-analysis of binomial data. Arch. Public Health.

[37] Clopper C.J., Pearson E.S. (1934). The Use of Confidence or Fiducial Limits Illustrated in the Case of the Binomial. Biometrika.

[38] Higgins J.P.T., Whitehead A., Simmonds M. (2011). Sequential methods for random-effects meta-analysis. Stat. Med..

[39] Sathish S., Chidambaram P., Uma A. (2021). Prevalence of parasites in tilapia farms and their management practices in Tamil Nadu. India J. Entomol. Zool. Stud..

[40] Zhang Y., Gong Q., Lv Q., Qiu Y., Wang Y., Qiu H., Guo X., Gao J., Chang Q., Wang C. (2020). Prevalence of *Clonorchis sinensis* infection in fish in South-East Asia: A systematic review and meta-analysis. J. Fish Dis..

[41] Gu E.D., Yu D.F., Yang X.Y., Xu M., Wei H., Luo D., Mu D.X., Hu Y.C. (2019). Tilapia fisheries in Guangdong Province, China: Socio-economic benefits, and threats on native ecosystems and economics. Fish Manag. Ecol..

[42] Rabie G., Ahlem M., Mehanna S. (2021). Reproductive dynamics of the redbelly tilapia (*Tilapia zillii* gervais, 1848) in ayata lake as a ramsar site in south-eastern algeria. Egypt. J. Aquat. Biol. Fish..

[43] Namaga W.M., Yahaya B., Salam M.A. (2020). Proximate composition of male and female African catfish (*Clarias gariepinus*) and tilapia (*Tilapia zilli*) in Jega river, Kebbi state, Nigeria. J. Fish Lives Vet. Scien..

[44] Guerrero R.D., Guerrero L.A. (2017). Preliminary studies on the breeding and culture of the black-chin tilapia (*Sarotherodon melanotheron*) in freshwater ponds. Asia Life Sci..

[45] Sparks J.S., Stiassny M.L.J. (2010). A new species of *Ptychochromis* from northeastern Madagascar (Teleostei: *Cichlidae*), with an update phylogeny and revised diagnosis for the genus. Zootaxa.

[46] Pérez-Ponce De León G., Lagunas-Calvo O., García-Prieto L., Briosio-Aguilar R., Aguilar-Aguilar R. (2018). Update on the distribution of the co-invasive *Schyzocotyle acheilognathi* (*Bothriocephalus acheilognathi*), the Asian fish tapeworm, in freshwater fishes of Mexico. J. Helminthol..

[47] Ahmad F., Fazili K.M., Sofi O.M., Sheikh B.A., Sofi T.A. (2018). Distribution and pathology caused by *Bothriocephalus acheilognathi* Yamaguti, 1934 (Cestoda: *Bothriocephalidae*): A review. Rev. Vet..

[48] Molnár K., Buchmann K., Székely C. (2019). Phylum Nematoda. Fish Diseases and Disorders.

[49] León-Règagnon V., Osorio-Sarabia D., García-Prieto L., Lamothe-Argumedo R., Bertoni-Ruiz F., Oceguera-Figueroa A. (2005). New host records of the nematode *Gnathostoma* sp. in Mexico. Parasitol. Int..

[50] Leroy J., Cornu M., Deleplancque A.S., Loridant S., Dutoit E., Sendid B. (2017). Sushi, ceviche and gnathostomiasis—A case report and review of imported infections. Travel Med. Infect Dis..

[51] Macpherson C.N.L. (2005). Human behaviour and the epidemiology of parasitic zoonoses. Int. J. Parasitol..

[52] Ogata K., Nawa Y., Akahane H., Camacho S.D., Lamothe-Argumedo R., Cruz-Reyes A. (1998). Short Report: Gnathostomiasis In Mexico. Am. J. Trop. Med. Hyg..

[53] Williams M., Hernandez-Jover M., Shamsi S. (2020). Fish substitutions which may increase human health risks from zoonotic seafood borne parasites: A review. Food Control..

[54] Burton B., Clint C., Thomas O. (2019). General Characteristics of the Trematoda. Human Parasitology.

[55] Kaleem O., Bio Singou Sabi A.F. (2020). Overview of aquaculture systems in Egypt and Nigeria, prospects, potentials, and constraints. Aquac. Fish..

[56] Squire S.A., Ryan U. (2017). *Cryptosporidium* and *Giardia* in Africa: Current and future challenges. Parasites Vectors.

[57] La Organización de las Naciones Unidas para la Alimentación y la Agricultura (FAO) World Food and Agriculture—Statistical Pocketbook World Food and Agriculture—Statistical Pocketbook 2018. https://www.fao.org/documents/card/es/c/ca6463en/.

[58] Huang S., He Y. (2019). Management of China’s capture fisheries: Review and prospect. Aquac. Fish..

[59] Chai J.Y., Jung B.K. (2017). Fishborne zoonotic heterophyid infections: An update. Food Waterborne Parasitol..

[60] Webster J.P., Molyneux D.H., Hotez P.J., Fenwick A. (2014). The contribution of mass drug administration to global health: Past, present and future. Philos. Trans. R. Soc. B Biol. Sci..

[61] Betson M., Alonte A.J., Ancog R.C., Aquino A.M., Belizario Jr V.Y., Bordado A.M., Clark J., Corales M.C., Dacuma M.G., Divina B.P. (2020). Zoonotic transmission of intestinal helminths in southeast Asia: Implications for Control and Elimination. Advances in Parasitology.

[62] Martínez Cruz J.M., Bravo Zamudio R., Aranda Patraca A., Martínez Marañón R. (1989). La gnatostomiasis en méxico. Salud Publica Mex..

[63] McCarthy J., Moore T.A. (2000). Emerging helminth zoonoses. Int. J. Parasitol..

[64] Diario Oficial de la Federación (DOF) (2009). NORMA Oficial Mexicana NOM-242-SSA1-2009, Productos y Servicios. Productos de la pesca frescos, refrigerados, congelados y procesados. Especificaciones sanitarias y métodos de prueba. Norma Of Mex. https://dof.gob.mx/normasOficiales/4295/salud2a/salud2a.htm.

[65] Tsiodras S., Kelesidis T., Kelesidis I., Bauchinger U., Falagas M.E. (2008). Human infections associated with wild birds. J. Infect..

[66] Adams A.M., Murrell K.D., Cross J.H. (1997). Parasites of fish and risks to public health. Rev. Sci. Tech..

[67] Pulido-Murillo E.A., Furtado L.F.V., Melo A.L., Rabelo É.M.L., Pinto H.A. (2018). Fishborne zoonotic trematodes transmitted by *Melanoides tuberculata* snails, Peru. Emerg. Infect. Dis..

[68] Vicente Pardo J.M. (2016). Anisakis and Disease as an Occupational Disease. Med. Segur. Trab..

[69] Arafa W.M., Hassan A.H.A., Mahrous L.N., Abdel-Ghany A.E., Aboelhadid S.M. (2019). Occurrence and molecular characterization of zoonotic *Anisakis simplex* sensu stricto and *Anisakis pegreffii* larvae in retail-marketed fish. J. Food Saf..

[70] Herman J.S. (2009). Gnathostomiasis Acquired by British Tourists in Botswana. Emerg. Infect. Dis..

[71] Violante-González J., García-Varela M., Rojas-Herrera A., Guerrero S.G. (2009). Diplostomiasis in cultured and wild tilapia *Oreochromis niloticus* in Guerrero State, Mexico. Parasitol. Res..

[72] Behringer D.C., Karvonen A., Bojko J. (2018). Parasite avoidance behaviours in aquatic environments. Philos. Trans. R. Soc. B Biol. Sci..

[73] Scholz T., Salgado-Maldonado G. (2000). The introduction and dispersal of *Centrocestus formosanus* (Nishigori, 1924) (Digenea: *Heterophyidae*) in Mexico: A review. Am. Midl. Nat..

[74] Umadevi K., Madhavi R. (2006). The life cycle of *Haplorchis pumilio* (Trematoda: *Heterophyidae*) from the Indian region. J. Helminthol..

[75] Amaechi C.E. (2015). Prevalence, intensity and abundance of endoparasites in *Oreochromis niloticus* and *Tilapia zilli* (Pisces: *Cichlidae*) from Asa Dam, Ilorin, Nigeria. UNED Res. J..

[76] Simon-Oke I.A. (2017). Diversity, intensity and prevalence of parasites of cichlids in polluted and unpolluted sections of Eleyele Dam, Ibadan, Nigeria. UNED Res. J..

[77] Yera H., Kuchta R., Brabec J., Peyron F., Dupouy-Camet J. (2013). First identification of eggs of the Asian fish tapeworm *Bothriocephalus acheilognathi* (Cestoda: Bothriocephalidea) in human stool. Parasitol. Int..

[78] Zhi T., Huang C., Sun R., Zheng Y., Chen J., Xu X., Brown C.L., Yang T. (2020). Mucosal immune response of Nile tilapia *Oreochromis niloticus* during *Gyrodactylus cichlidarum* infection. Fish. Shellfish Immunol..

[79] Papkou A., Gokhale C.S., Traulsen A., Schulenburg H. (2016). Host-parasite coevolution: Why changing population size matters. Zoology.

[80] Mutengu C., Mhlanga W., Mupangwa J.F. (2018). Occurrence of *Clinostomum* metacercariae in *Oreochromis mossambicus* from Mashoko Dam, Masvingo Province, Zimbabwe. Scientifica.

[81] Cortés D.A., Dolz G., Zúñiga J.J.R., Rocha A.E.J., Alán D.L. (2010). *Centrocestus formosanus* (Opisthorchiida: *Heterophyidae*) como causa de muerte de alevines de tilapia gris *Oreochromis niloticus* (Perciforme: *Cichlidae*) en el Pacífico seco de Costa Rica. Rev. Biol. Trop..

[82] Lun Z.R., Gasser R.B., Lai D.H., Li A.X., Zhu X.Q., Yu X.B., Fang Y.Y. (2005). Clonorchiasis: A key foodborne zoonosis in China. Lancet Infect. Dis..

[83] Dzikowski R., Diamant A., Paperna I. (2003). Trematode metacercariae of fishes as sentinels for a changing limnological environment. Dis. Aquat. Organ..

[84] Pinto H.A., Mati V.L.T., Melo A.L. (2014). Metacercarial infection of wild nile tilapia (*Oreochromis niloticus*) from Brazil. Sci. World J..

[85] Eissa A.E., Attia M.M., Elgendy M.Y., Ismail G.A., Sabry N.M., Prince A. (2021). *Streptococcus*, *Centrocestus formosanus* and *Myxobolus tilapiae* concurrent infections in farmed Nile tilapia (*Oreochromis niloticus*). Microb. Pathog..

[86] Yimer E. (2000). Preliminary survey of parasites and bacterial pathogens of fish at Lake Ziway. Ethiop. J. Sci..

[87] Echi P.C., Eyo J.E., Okafor F.C. (2009). Co-Parasitism And Morphometrics Of Three Clinostomatids (Digenea: *Clinostomatidae*) In *Sarotherodon melanotheron* From A Tropical Freshwater Lake. Anim. Res. Int..

[88] Onyedineke E.N., Obi U., Ofoegbu P.U., Ukogo I. (2010). Helminth Parasites of some Freshwater Fish from River Niger at Illushi, Edo State, Nigeria. J. Am. Sci..

[89] Roche D.G., Leung B., Mendoza Franco E.F., Torchin M.E. (2010). Higher parasite richness, abundance and impact in native versus introduced cichlid fishes. Int. J. Parasitol..

[90] Gulelat Y., Yimer E., Asmare K., Bekele J. (2013). Study on parasitic helminths infecting three fish species from Koka reservoir, Ethiopia. SINET Ethiop. J. Sci..

[91] Amare A., Alemayehu A., Aylate A. (2014). Prevalence of internal parasitic helminthes infected *Oreochromis niloticus* (Nile Tilapia), *Clarias gariepinus* (African Catfish) and *Cyprinus carpio* (Common Carp) in Lake Lugo (Hayke), Northeast Ethiopia. J. Aquac. Res. Dev..

[92] Okoye I.C., Abu S.J., Obiezue N.N.R., Ofoezie I.E. (2014). Prevalence and seasonality of parasites of fish in Agulu Lake, Southeast, Nigeria. Afr. J. Biotechnol..

[93] Walakira J., Akoll P., Engole M., Sserwadda M., Nkambo M., Namulawa V. (2014). Common fish diseases and parasites affecting wild and farmed tilapia and catfish in central and western Uganda. Uganda. J. Agric. Sci..

[94] Bekele J., Hussien D. (2015). Prevalence of Internal Parasites of *Oreochromis niloticus* and *Clarias gariepinus* Fish Species in Lake Ziway, Ethiopia. J. Aquac. Res. Dev..

[95] Reshid M., Adugna M., Tsegaye Redda Y., Awol N., Teklu A. (2015). A Study of *Clinostomum* (Trematode) and *Contracaecum* (Nematode) Parasites Affecting *Oreochromis Niloticus* in Small Abaya Lake, Silite Zone, Ethiopia. J. Aquac. Res Dev..

[96] Sèdogbo M.H., Zannou B.T., Siko J.E., Tossavi N.D., Togla I., Fiogbé E.D. (2019). Faune des métazoaires parasites de *Clarias gariepinus* (Clariidae) et de *Oreochromis niloticus* (Cichlidae), deux poissons des whédos du delta supérieur du fleuve Ouémé au sud du Bénin. Int. J. Biol. Chem. Sci..

[97] Adugna M., Fishery N., Life A. (2020). The Prevalence of Fish Parasites of Nile Tilapia (*Oreochromis niloticus*) in Selected Fish farms, Amhara Regional State. Ethiop. J. Agric. Sci..

[98] Otachi E., Magana A., Jirsa F., Fellner-Frank C. (2014). Parasites of commercially important fish from Lake Naivasha, Rift Valley, Kenya. Parasitol. Res..

[99] Otachi E., Szostakowska B., Jirsa F., Fellner-Frank C. (2015). Parasite communities of the elongate tigerfish *Hydrocynus forskahlii* (Cuvier 1819) and redbelly tilapia *Tilapia zillii* (Gervais 1848) from Lake Turkana, Kenya: Influence of host sex and size. Acta Parasitol..

[100] Garrido-Olvera L., Benavides-González F., Rábago-Castro J.L., Pérez-Castañeda R., García-Prieto L. (2017). Endohelminths of Fishes of Commercial Importance from Vicente Guerrero Reservoir, Tamaulipas, Mexico. Comp. Parasitol..

[101] Davidovich N., Tedesco P., Caffara M., Yasur-Landau D., Gustinelli A., Drabkin V. (2022). Morphological description and molecular characterization of *Contracaecum* larvae (Nematoda: *Anisakidae*) parasitizing market-size hybrid tilapia (*Oreochromis aureus* x *Oreochromis niloticus*) and red drum (*Sciaenops ocellatus*) farmed in Israel. Food Waterborne Parasitol..

[102] Kang L., Hedegaard Clausen J., Murrell D., Liu L., Dalsgaard A. (2013). Risks for fishborne zoonotic trematodes in Tilapia production systems in Guangdong province, China. Vet. Parasitol..

[103] Wilson J.R., Saunders R.J., Hutson K.S. (2019). Parasites of the invasive tilapia *Oreochromis mossambicus*: Evidence for co-introduction. Aquat. Invasions.

[104] Mosqueda Cabrera M.Á., Miranda E.S., Calderón L.C., Ortiz Nájera H.E. (2009). Finding advanced third-stage larvae of *Gnathostoma turgidum* Stossich, 1902 in Mexico from natural and experimental host and contributions to the life cycle description. Parasitol. Res..

[105] Thien P., Dalsgaard A., Thanh B.N., Olsen A., Murrell K.D. (2007). Prevalence of fishborne zoonotic parasites in important cultured fish species in the Mekong Delta, Vietnam. Parasitol. Res..

[106] Díaz M.T., Hernandez L.E., Bashirullah A.K. (2008). Studies on the life cycle of *Haplorchis pumilio* (Looss, 1896) (Trematoda: Heterophyidae) in Venezuela. Rev. Cient. la Fac. Ciencias Vet la Univ. del Zulia.

[107] Thien P., Dalsgaard A., Thanh Nhan N., Olsen A., Murrell K.D. (2009). Prevalence of zoonotic trematode parasites in fish fry and juveniles in fish farms of the Mekong Delta, Vietnam. Aquaculture.

[108] Chi T.T., Murrell K.D., Mausen H., Khue N.V., Dalsgaard A. (2009). Fishborne zoonotic trematodes in raw fish dishes served in restaurants in nam dinh province and hanoi, vietnam. J. Food Prot..

[109] Lobna S.M.A., Metawea Y.F., Elsheikha H.M. (2010). Prevalence of heterophyiosis in Tilapia fish and humans in Northern Egypt. Parasitol Res..

[110] Thi Phan V., Kjær Ersbøll A., Quang Bui T., Thi Nguyen H., Murrell D., Dalsgaard A. (2010). Fish-Borne Zoonotic Trematodes in Cultured and Wild-Caught Freshwater Fish from the Red River Delta, Vietnam. Vector-Borne Zoonotic Dis..

[111] Thi Phan V., Kjær Ersbøll A., Nguyen K.V., Madsen H., Dalsgaard A. (2010). Farm-level risk factors for Fish-borne zoonotic trematode infection in integrated Small-scale fish farms in Northern Vietnam. PLoS Negl. Trop. Dis..

[112] Van De N., Le T.H., Murrell K.D. (2012). Prevalence and intensity of fish-borne zoonotic trematodes in cultured freshwater fish from rural and Urban Areas of Northern Vietnam. J. Parasitol..

[113] Hegazi M.A., Abo-elkheir O.I. (2014). Encysted Metacercariae of Family Heterophyidae in Infected Fish in Dakahlia Governorate, an Endemic Focus in Egypt. J. Egypt. Soc. Parasitol..

[114] Kopolrat K., Sithithaworn P. (2015). Susceptibility, metacercarial burden, and mortality of juvenile silver barb, common carp, mrigal, and tilapia following exposure to *Haplorchis taichui*. Parasitol. Res..

[115] Madsen H., Dung B.T., The D.T., Viet N.K., Dalsgaard A., Van P.T. (2015). The role of rice fields, fish ponds and water canals for transmission of fish-borne zoonotic trematodes in aquaculture ponds in Nam Dinh Province, Vietnam. Parasites Vectors.

[116] Ojwala R.A., Otachi E.O., Kitaka N.K. (2018). Effect of water quality on the parasite assemblages infecting Nile tilapia in selected fish farms in Nakuru County, Kenya. Parasitol. Res..

[117] Scholz T., Šimková A., Razanabolana J.R., Kuchta R. (2018). The first record of the invasive Asian fish tapeworm (*Schyzocotyle acheilognathi*) from an endemic cichlid fish in Madagascar. Helminthologia.

