# Supplementary material for: Prevalence of Parasitic Infections with Zoonotic Potential in Tilapia: A Systematic Review and Meta-Analysis
Source: Animals (Basel). 2022 Oct 17;12(20):2800. doi: 10.3390/ani12202800 (PMC9597807; doi:10.3390/ani12202800)
Supplement: Supplementary file 1 [file animals-12-02800-s001.zip › animals-1882507-supplementary materials.pdf]

Table S1. Parasites of tilapia with zoonotic potential according with the available scientific literature.

Authors	Parasite	Taxonomic group	Infective stage	Cichlid host/Anatomical region
(19,29,83-85)	*Centrocestus* spp.	Trematoda	Metacercariae	*Oreochromis* spp.<br>*Sarotherodon* spp.
(28,86-97)	*Clinostomum* spp.	Trematoda	Metacerariae	*Oreochromis* spp.<br>*Sarotherodon* spp.<br>*Tilapia* spp.<br>*Vieja* pp.
(12,21,86,89–91,94,95,97–101)	*Contracaecum* spp.	Nematoda	L3 Larva	*Oreochromis* spp.<br>*Tilapia* spp.<br>*Vieja* pp.
(23)	*Cryptosporidium* spp.	Protozoa	Trophozoites	*Oreochromis* spp.
(102)	Echinostomatidae	Trematoda	Metacercariae	*Oreochromis* spp.
(103)	*Echinostoma* spp.	Trematoda	Metacercariae	*Oreochromis* spp.
(91,94)	*Eustrongylides* spp.	Nematoda	L3 Larva	*Oreochromis* spp.
(23)	*Giardia* spp.	Protozoa	Trophozoites	*Tilapia* spp.
(20,49,104)	*Gnathostoma* spp.	Nematoda	L3 Larva	*Oreochromis* spp.
(13,15-17,19,33,83,105–115**)**	*Haplorchis* spp.	Trematoda	Metacercariae	*Oreochromis* spp.<br>*Sarotherodon* spp.<br>*Tilapia* spp.
(34,98,109,113,116)	*Heterophyes* spp.	Trematoda	Metacercariae	*Oreochromis* spp.<br>*Tilapia* spp.
(102)	Heterophydae	Trematoda	Metacercariae	*Oreochromis* spp.
(32)	*Opisthorchis* spp.	Trematoda	Metacercariae	*Oreochromis* spp.
(16,33,113)	*Phagicola* spp.	Trematoda	Metacercariae	*Tilapia* spp.

(13,17)	*Procerovum* spp.	Trematoda	Metacercariae	*Oreochromis* spp.<br>*Tilapia* spp.
(16,33,109,113)	*Pygidiopsis* spp.	Trematoda	Metacercariae	*Tilapia* spp.
(117)	*Schyzocotyle* spp.	Cestoda	Plerocercoid larva	*Ptychochromis* spp.
(26)	*Vermoameba* spp.	Amoeba	Trophozoites	*Oreochromis* spp.
